# Long Non-coding RNAs and Their Biological Roles in Plants

**DOI:** 10.1016/j.gpb.2015.02.003

**Published:** 2015-04-30

**Authors:** Xue Liu, Lili Hao, Dayong Li, Lihuang Zhu, Songnian Hu

**Affiliations:** 1 CAS Key Laboratory of Genome Sciences and Information, Beijing Institute of Genomics, Chinese Academy of Sciences, Beijing 100101, China; 2 State Key Laboratory of Plant Genomics and National Center for Plant Gene Research, Institute of Genetics and Developmental Biology, Chinese Academy of Sciences, Beijing 100101, China

**Keywords:** Long non-coding RNA (lncRNA), Long non-coding natural antisense transcripts (lncNATs), Epigenetic, Small RNA, MicroRNA, Target mimicry

## Abstract

With the development of genomics and bioinformatics, especially the extensive applications of high-throughput sequencing technology, more transcriptional units with little or no protein-coding potential have been discovered. Such RNA molecules are called non-protein-coding RNAs (npcRNAs or ncRNAs). Among them, long npcRNAs or ncRNAs (lnpcRNAs or lncRNAs) represent diverse classes of transcripts longer than 200 nucleotides. In recent years, the lncRNAs have been considered as important regulators in many essential biological processes. In plants, although a large number of lncRNA transcripts have been predicted and identified in few species, our current knowledge of their biological functions is still limited. Here, we have summarized recent studies on their identification, characteristics, classification, bioinformatics, resources, and current exploration of their biological functions in plants.

## Introduction

As a class of RNAs that have no or little protein-coding potential, the mechanism underlying the functions of non-protein coding RNAs (ncRNAs or npcRNAs) is a fascinating area of research [1]. The recent wide applications of the high-throughput RNA-sequencing (RNA-seq) approaches have facilitated the identification of thousands of novel ncRNAs (or npcRNAs) in many organisms, such as humans, animals, and plants [2–6]. The ncRNAs are a heterogeneous group of RNA molecules, which can be classified in different ways according to their location, length, and biological functions [1,7–10].

The canonical ncRNAs such as ribosomal RNAs (rRNAs) and transfer RNAs (tRNAs) were discovered earlier owing to their important functions in protein synthesis in all living organisms. Small RNAs (sRNAs), for instance, small nucleolar and small nuclear RNAs (snoRNAs and snRNAs) are found in specific cellular locations, which can function through modification of other RNAs (*e.g.*, rRNAs and tRNAs) and processing of pre-mRNA [5]. Besides RNAs with specific functions, other ncRNAs are mainly classified based on the length of their mature products. Small ncRNAs of 20−30 nucleotides (nt) in length are mainly microRNAs (miRNAs) and small interfering RNAs (siRNAs), commonly found as transcriptional and translational regulators [11]. Medium ncRNAs of 50−200 nt in length and long ncRNAs (lncRNAs) with size beyond 200 nt are usually involved in other processes, such as splicing, gene inactivation, and translation [1,10,12,13]. To date, the best-characterized ncRNAs are sRNAs [14,15].

As mentioned above, lncRNAs are arbitrarily defined as RNA transcripts that contain > 200 nt but lack protein-coding potential [16]. lncRNAs are transcribed by RNA polymerase II or III, and additionally, by polymerase IV/V in plants [17–19]. They are processed by splicing or non-splicing, polyadenylation or non-polyadenylation, and can be located in the nucleus or cytoplasm. Functional analyses of lncRNAs have shown that they are potent *cis-* and *trans*-regulators of gene transcription, and act as scaffolds for chromatin-modifying complexes. As potent regulatory components involved in gene regulation from various aspects, lncRNAs can exert their effects during tissue development and in response to external stimuli [20]. lncRNAs are classified primarily based on four major features, namely, genomic location, functions exerted on DNA or RNA, functioning mechanisms, and targeting mechanisms [12].

Although lncRNAs have received more attention in recent years, the research in this field is still in its infancy. Thus far, only a few lncRNAs have been sufficiently described [21–23]. In particular, research in this area in plants is far behind that in humans and animals [9,24,25]. Nonetheless, studies available suggest that plant lncRNAs exert regulatory functions similar to those in animals [9,24]. In order to gain a better understanding of recent progress in the research of plant lncRNAs, we provide a brief overview on their discovery and functional analyses in the following context.

## Discovery of lncRNAs in plants

Novel ncRNAs can be detected and discovered by both experimental and computational screenings [26]. Genome-wide approaches used for transcriptomic analyses such as microarrays and RNA sequencing in model organisms have revealed that non-protein coding transcripts occupy most of the eukaryote transcriptome, much higher than that previously believed [7,27–33]. Especially, next-generation sequencing (NGS)-based technology provides us with a more complex perspective and a much closer and complete view of the RNA world. lncRNAs have been discovered in yeast and other higher eukaryotes [34–36]. For instance, genome-wide analyses have discovered more than 50,000 lncRNAs in the human genome [35,37–39].

About 6480 lncRNAs were identified from 200 *Arabidopsis thaliana* transcriptomic data sets, with either organ-specific or stress-induced expression profiles [7]. Wang et al. discovered 37,238 long non-coding natural antisense transcripts (lncNATs) in *A. thaliana*, with antisense transcripts associated with 70% of annotated mRNAs [28]. Using a strand-specific RNA sequencing approach, Zhu et al. [40] identified lncRNAs in *A. thaliana* induced by *Fusarium oxysporum* infection. Results showed that antisense transcripts existed in about 20% of the annotated genes, and most newly-identified transcriptionally-active regions (TARs) were adjacent to or located as an extension of the annotated genes. Besides poly(A)^+^ lncRNAs, lncRNAs without poly(A) tails (poly(A)^−^ lncRNAs) were also identified in humans [41]. In plants, the presence of poly(A)^−^ lncRNAs was revealed in seedlings of *A. thaliana* under different stress conditions using RNA-seq [42]. Compared to poly(A)^+^ lncRNAs, poly(A)^−^ lncRNAs are shorter, have lower expression, and are more specific in response to stresses.

Combining both computational and experimental analyses, Xin et al. [43] identified 125 putative stress responsive lncRNAs in wheat. These lncRNAs were tissue-specific and can be induced by powdery mildew infection and heat stress. lncRNAs were also reported in maize. Li et al. [27] identified 20,163 putative lncRNAs in maize by integrating the available EST data, annotated information of maize genome, and RNA-seq datasets obtained from 30 different experiments. By comparing these putative lncRNAs to a comprehensive set of maize sRNAs, they found that more than 90% of these lncRNAs are potential precursors of sRNAs, while only 1704 are high-confidence lncRNAs. It is of note that half of the high-confidence lncRNAs were tissue specific, as supported by the tissue-specific H3K27me3 heterochromatin epigenetic mark. In addition, Zhang et al. [44] performed strand-specific RNA sequencing of rice anthers, pistils, seeds, and shoots. In combination with the analysis of other available rice RNA-seq datasets, they systematically identified 2224 lncRNAs from rice and showed that rice lncRNAs were highly tissue-specific or stage-specific. Studies integrating strand-specific RNA sequencing and sRNA sequencing data were also reported in detecting NATs in rice under normal and different stress conditions. In total 2292 putative *cis*-NATs were shown to be expressed, among which 503 *cis*-NATs were expressed under specific conditions [45]. In addition, sRNAs were also detected from their corresponding overlapping regions.

Besides lncRNAs identified in the model plant *Arabidopsis*, rice, and maize, Qi et al. identified 584 lncRNAs that were responsive to simulated drought stress in foxtail millet by using a deep transcriptomic sequencing approach [46]. Ye et al. identified many endogenous target mimics (eTM, a class of lncRNAs which are complementary to miRNAs as decoy RNAs to prevent miRNAs from binding to their authentic targets) and phased siRNA (phasiRNA, phased secondary siRNAs which function in *trans* to suppress the expression of target transcripts)-producing loci (*PHAS*) genes in soybean [47]. They found that miRNAs potentially regulate lipid metabolism-related genes and trigger the production of phasiRNAs from *PHAS* genes, although some of these miRNAs can be further regulated by eTMs [47]. Additional efforts to identify more novel lncRNAs have also been exerted in other plants such as peach [48], populus [49,50], and *Brassica rapa* [51] by employing RNA sequencing strategy.

Other than direct transcriptomics analysis, chromatin signature-based approach was also used to define TARs. K4–K36 domain is usually used to define TARs, since active promoter that is marked by H3K4me3 usually combines with TARs that are marked by H3K36me3. In humans and mice, many lncRNAs were identified by the presence of K4–K36 domains in the intergenic regions [4,35]. However, this approach has only been adopted in *Arabidopsis* among model plants. Information on chromatin states should be obtained in other model plants and crops to assist the identification of TARs in the future [52,53].

## Plant lncRNAs as precursors of miRNAs and other sRNAs

As an emerging class of riboregulators, lncRNAs either act directly or are processed to shorter ncRNAs for functioning [54]. Some lncRNAs are primary transcripts of small regulatory RNAs such as miRNAs and siRNAs. Similar to protein-coding genes and some lncRNAs, primary transcripts of miRNA (pri-miRNA) genes are transcribed by RNA polymerase II (Pol II) [55]. In contrast to vertebrates and flies, miRNAs in plants are minor constituents because plants have more complex small regulatory RNA pools. Such complexity of sRNA pools in plants can be exemplified by the presence of plant-specific RNA polymerase IV/V (Pol IV/Pol V)-dependent siRNAs and secondary endogenous siRNAs [56]. Biogenesis pathway of the Pol IV/Pol V-dependent siRNAs also produces a plant-specific class of lncRNAs called the Pol IV/V-dependent lncRNAs, which are required for RNA-directed DNA methylation (RdDM) [20].

Analysis of the full-length cDNA databases led to the identification of numerous 24-nt siRNAs that were matched with five lncRNAs including npc34, npc351, npc375, npc520, and npc523 in *Arabidopsis*. Most siRNAs derived from these five lncRNAs are mapped to both strands of the lncRNA region, suggesting that these lncRNAs are siRNA precursors [1]. Mapping sRNA present in databases [57] to the complete collection of 76 lncRNAs [54] revealed that 34 lncRNAs are potential precursors of sRNAs. For example, miRNAs miR869a and miR160c mature from npc83 and npc521, respectively. Based on sRNA sequencing and degradome sequencing data in *Arabidopsis*, Ma et al. [58] identified 43 regions that have the potential to form highly-complementary long-stem structures, which can be potentially recognized by Dicer-like 1 (DCL1) for further cropping, suggesting that these regions may function as sRNA precursors [58]. It is noteworthy that Lauressergues et al. recently discovered that peptides can be encoded by the non-coding regions of miRNA precursors, indicating that some lncRNAs may still possess coding potential [59,60].

## Plant lncRNAs as miRNA target mimics

Target mimicry was first found in plants, rising as a novel mechanism for regulating miRNA functions [61]. During target mimicry, interactions between miRNAs and their authentic targets are blocked by the binding of decoy RNAs to miRNAs via partially-complementary sequences [61,62]. Recently, competing endogenous RNAs (ceRNAs) with similar mechanisms were also identified in human and animal cells, indicating that inhibition of miRNA activity by target mimicry may be a widespread phenomenon [38,63,64].

As an endogenous lncRNA, *Induced by Phosphate Starvation 1* (*IPS1*) was first identified in *A. thaliana*, which functioned as an eTM of miR399 [61,62]. Pairing with a three-nucleotide bulge, *IPS1* binds to miR399 and destroys the miR399-mediated cleavage of its target genes. Thus, *IPS1* interferes with the binding of ath-miR399 to its authentic targets as a decoy [61]. Genome-wide analyses have identified some candidate eTMs in several plant species with completely-sequenced genomes [65–67]. However, most predictions of eTMs were mainly performed on annotated genes. eTMs for 20 miRNAs conserved in *Arabidopsis* and rice were systematically identified in intergenic or non-coding gene regions by Wu and colleagues [62]. They show that different eTMs can bind to the same miRNA and the binding sites were well conserved among eTMs, while sequences flanking the miRNA binding sites varied a lot. Using agroinfiltration-based transient expression assay, they identified the important regulatory roles of functional target mimics for miR160 and miR166 in plant development and validated the effectiveness of eTMs for three other miRNAs including ath-miR156, ath-miR159, and ath-miR172 [62].

Target mimicry effects can be induced by both endogenous and engineered artificial miRNA TMs [62,68,69]. Therefore, in addition to their important biological significance, discovery of miRNA target mimics has provided an alternative method for functional studies on miRNAs. For instance, artificial TMs imported into transgenic plants were capable of attenuating the functions of corresponding miRNAs [65,68,70].

## Plant lncRNAs and vernalization

Flowering time is one of the most important adaptive traits to ensure the transition of reproductive growth and development that occurs under favorable conditions during a plant’s life cycle [71]. Vernalization is an important mechanism controlling flowering in some plant species that grow in a vegetative state during the cold winter seasons and begin to flower in the warmer spring [72,73]. Vernalization is the best-studied regulatory process in plants that is known to involve lncRNAs, primarily in the regulation of *FLOWERING LOCUS C* (*FLC*) gene [74,75].

*FLC* is a key regulator of flowering time in *A. thaliana* [76], which acts as a repressor to inhibit flowering under cold temperature [76]. *FLC* gene is located at a complex locus. Recent studies have shown that at least two types of lncRNAs are present in this locus*.* A group of long antisense RNAs, called COLD INDUCED LONG ANTISENSE INTRAGENIC RNAs (COOLAIR) are transcribed in antisense orientation in relation to *FLC* [23,77,78], whereas another lncRNA *COLD ASSISTED INTRONIC NONCODING* RNA (*COLDAIR*), is transcribed from the intron of *FLC* gene in the sense orientation [22]. Both lncRNAs can help recruit PHD-PRC2 complex to enable histone modifications of *FLC* via epigenetic regulation. Considering that there have been several excellent review articles on this topic [79–81], we do not go into too much detail in this paper.

## Plant lncRNAs and fertility

Rice is an important crop as well as an important model organism. Breeding of hybrid rice is one of the evolutionary applications of heterosis in agriculture and male sterility lines are essential for this process. However, little is known about the regulatory genes and molecular mechanisms underlying plant male sterility. Ding et al. and Zhou et al. [82,83] cloned the gene controlling photoperiod-sensitive genetic male sterility (PSMF) independently, and found that the cloned gene was a lncRNA. However, the action modes of this gene are not consistent in the two studies. Ding et al. [82] suggested that sufficient amount of the lncRNA, which they termed as *long day* (*LD*)*-specific male-fertility-associated RNA* (*LDMAR*), is necessary for rice fertility under LD conditions. The transcription level of *LDMAR* is reduced specifically under LD conditions, which results in programmed cell death (PCD) during rice anther development and causes male sterility [82]. They also identified a spontaneous point mutation, which led to alteration in RNA secondary structure and increased DNA methylation in its promoter region. Further investigation [84] by the same group showed that an siRNA, *Psi–LDMAR*, is produced in the promoter region of *LDMAR*. Overexpression of *Psi–LDMAR* induced RdDM in the promoter region of *LDMAR* and resulted in reduced expression of *LDMAR*. Zhou et al. [83] found that *P*/*TMS12-1* (another name for *LDMAR*) encodes a unique ncRNA, which produces a 21-nt sRNA, *osa-smR5864w*. A C-to-G point mutation present in *osa-smR5864w* may lead to loss-of-function of the sRNA, eventually resulting in the production of light- and temperature-sensitive male sterile rice [83]. The different explanations of the action mechanisms between the two groups illustrate the complex functions of lncRNAs. Therefore, further detailed mechanistic study is required.

## Plant lncRNAs and photomorphogenesis

Light is regarded as an important ecological factor to regulate almost all processes of growth and development in plants [85]. The mechanistic study of photomorphogenesis is one of the hotspots in plant molecular biology. The sophisticated regulatory processes of photomorphogenesis have been thoroughly elucidated, and many important mechanisms are well understood at the molecular level. However, the regulatory factors identified so far were mainly proteins. The involvement of lncRNAs in this process is yet to be explored and is an interesting area of research.

As aforementioned, Wang et al. had identified genome-wide lncNATs in model plant *A. thaliana* [28]. They focused on the roles of lncRNAs in response to light and identified 626 concordant and 766 discordant NAT pairs in *A. thaliana*, with many light-responsive lncNATs related to histone modifications. It would be very interesting to explore the functions of lncRNAs in phototropic responses. Deng et al. [86] identified and functionally characterized a novel 236-nt lncRNA, *HIDDEN TREASURE 1* (*HID1*), which is involved in the sophisticated photomorphogenic process. By screening their T-DNA insertion mutant collection, they identified a mutant named *hid1* later on, which exhibits a hypo-photomorphogenic phenotype under continuous red light (cR). The mutant results from the loss-of-function of the lncRNA gene *HID1*. Through detailed analyses, the authors discovered that *HID1* may function by regulating the expression of the transcription factor, *PHYTOCHROME-INTERACTING FACTOR 3* (*PIF3*), one of the key repressors in photomorphogenesis that modulates light response [86]. Genetic analyses showed that *HID1* could negatively regulate the expression of *PIF3* gene. *HID1* is located in the nucleus and can associate with chromatin and may bind directly to the promoter region of *PIF3* to repress its expression [86]. It is of note that *HID1* homologs are found in many plant species [86] and may possess conserved functions in different species. For instance, *OsHID1*, the rice homolog, can rescue the phenotype of *hid1* mutant in *A. thaliana* [86]. *HID1* is the first known lncRNA involved in photomorphogenesis, shedding light on the association of ncRNAs and light response in plants [86].

## Plant lncRNAs and phosphate homeostasis

**Figure 1:**
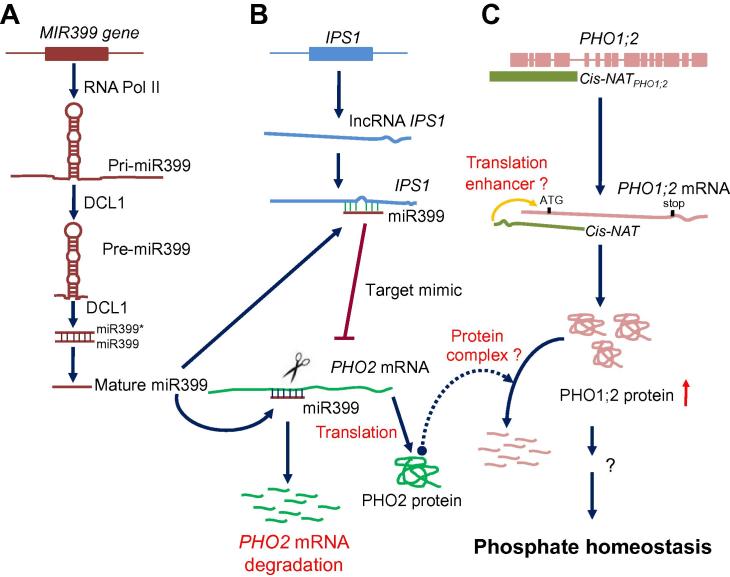
**The lncRNA-related regulatory networks for phosphate homeostasis in plants** **A.** miR399 can suppress the expression of its target gene, *PHOSPHATE2* (*PHO2*), to control phosphate homeostasis [91,92]. miR399 is encoded by *MIR399* genes, for which primary transcripts are lncRNAs. **B.** lncRNA *IPS1* is induced under phosphate deficiency and acts as a target mimic for miR399 to regulate the phosphate homeostasis [61]. **C.***Cis*-natural antisense RNA, *cis-NAT_PHO1;2,_* can act as a translational enhancer for the expression of its sense gene, *PHOSPHATE1;2* (*PHO1;2*), to control phosphate homeostasis in rice [89].

Phosphate is an essential mineral nutrient for plant growth and development [87,88]. Several lines of evidence have suggested that lncRNAs are involved in the phosphate homeostasis [61,89]. First, some miRNAs have been reported to exert effects in regulating phosphate homeostasis [88,90,91]. The well-studied miR399 [91,92] can suppress the expression of its target gene, *PHOSPHATE2* (*PHO2*), which encodes a ubiquitin-conjugating E2 enzyme (Figure 1**A**). PHO2 can interact with PHOSPHATE1 (PHO1), a membrane protein involved in phosphate loading to the xylem and a key regulator for phosphate homeostasis conserved in plants [93], to control phosphate homeostasis [92]. Since plant miRNAs are mainly encoded by lncRNAs [10,15], involvement of miRNAs in phosphate homeostasis is indicative of the involvement of lncRNAs in phosphate homeostasis. In addition, the aforementioned eTM-type lncRNAs *IPS1* exemplifies the direct involvement of lncRNAs in phosphate homeostasis [61]. *IPS1* is induced under phosphate deficiency and acts as a target mimic for miR399 (Figure 1**B**) [61]. Jabnoune et al. reported another layer of regulation in plants. They found that in rice, the *cis*-natural antisense RNA, *cis-NAT_PHO1;2_*, can act as a translational enhancer for the expression of its sense gene, *PHOSPHATE1;2* (*PHO1;2*) (Figure 1**C**) [89], the functional ortholog of *PHO1* in *Arabidopsis* [94]. These findings reveal that there exists complex RNA regulatory network to control phosphate homeostasis in plants. Other lncRNAs related to phosphate homeostasis in tomato and rice are listed in Table 1, together with other important lncRNAs reported in plants [95–99].

## Plant lncRNAs and protein re-localization

*Early nodulin 40* (*ENOD40*) [100] is conserved in legumes [101,102] and is found in several non-legume species [103,104] as well. *ENOD40* participates in the regulation of symbiosis between bacteria or fungi and leguminous plants [104,105]. During symbiotic interaction, *ENOD40* expression is rapidly induced by rhizobia in the nodule primordium [106]. Although the underlying molecular mechanisms are not clear, *ENOD40* can play roles in transporting metabolites necessary for cell growth in non-symbiotic plants [105].

*ENOD40* encodes two short peptides but there lacks long open reading frame [107,108]. *ENOD40* exerts its biological activity directly by translation of these two short peptides in barrel medic (*Medicago truncatula*) [107], while in soybean, the two peptides of ENOD40 bind specifically to sucrose synthase, suggesting its role in sucrose utilization [108]. Five conserved domains in *ENOD40* mRNA are found in various leguminous and non-leguminous species. Notably, one structural domain conserved in *Enod40* is similar to the expansion segments in some structural RNAs [109]. Structural elements of *ENOD40* mRNA are much more conserved than the encoded short peptides, which suggest that the RNA structure determines the principal functions of *ENOD40*, whereas more diverse functions, revealed in a minority of plant families, are exerted by short peptides. This hypothesis has been proved in *M. truncatula* [110].

*M*. *truncatula* RNA-binding protein 1 (MtRBP1) directly interacts with *ENOD40* in mature nodules, where *ENOD40* is expressed at high levels [110]. MtRBP1 re-localizes from nuclear speckles to cytoplasmic granules with the aid of *ENOD40* during nodulation in *M. truncatula*. MtRBP1 protein could be found localized in the cytoplasm only when *ENOD40* was co-expressed in these cells. Induction of MtRBP1 relocalization was similarly achieved by *ENOD40* transcripts with the initial ATG mutated, indicating that *ENOD40*-encoded peptides are not involved in this activity. Hence, for the re-localization activity of MtRBP1, RNA structures, and not the encoded short peptides of *ENOD40*, are required.

## Plant lncRNAs and alternative splicing

Alternative splicing is an important regulatory layer in gene expression. Multiple variants of proteins or transcripts can be generated from a single gene via alternative splicing, thus increasing the complexity of proteome and transcriptome. Bardou et al. reported the involvement of lncRNA in alternative splicing in *Arabidopsis* [111]. They found that an lncRNA acts as an alternative splicing competitor (ASCO). The ASCO-lncRNA and the nuclear speckle RNA-binding protein (NSR) could form an alternative splicing regulatory module. *AtNSR* is mainly expressed in primary and lateral root meristems and regulates development of lateral roots. Transgenic plants overexpressing the ASCO-lncRNA exhibit an altered ability to form lateral roots, which is similar to the phenotypes of the double *Atnsr* mutants. AtNSRs interact with the ASCO-lncRNA *in vivo* and affect the splicing patterns of NSR-regulating mRNA targets. It seems that lncRNA can recruit the alternative splicing regulators to modulate the related processes [111,112].

## Plant lncRNAs and modulation of chromatin loop dynamics

Dynamic chromatin topology can affect the pattern of gene expression [113]. Ben Amor et al. identified 76 lncRNAs from the *Arabidopsis* full-length cDNA databases by using bioinformatics approach [1]. Among them, npc34, was renamed as *AUXIN REGULATED PROMOTER LOOP* (*APOLO*) in a recent study [114]. This intergenic lncRNA is encoded by a gene located about 5 kb upstream of *PINOID* (*PID*) and is transcribed by two RNA polymerases, RNA Pol II and Pol V [114]. It was reported that the dual *APOLO* transcription could control the chromatin loop dynamics to regulate the promoter activity of the neighbor *PID* gene [114], which is an important regulator of polar auxin transport [114,115]. The phytohormone auxin regulates expression of *APOLO* and *PID*. Exogenous auxin treatment can activate the demethylation of the *APOLO−PID* genomic region and the chromatin loop encompassing the promoter region of *PID*. When the loop is opened, RNA Pol II transcribes the two genes and the accumulation of both *PID* and *APOLO* RNAs is increased. Then, the *APOLO* transcripts produced by RNA Pol II gradually recruit the polycomb repressive complex 1 (PRC1) to close the loop. Then the *APOLO* transcripts produced by RNA Pol V are recruited by ARGONAUTE4 (AGO4) and trigger DNA methylation. Finally, the *APOLO* lncRNAs-mediated chromatin loop is reformed and *PID* expression is down-regulated [114]. It seems that the dual lncRNA transcription influencing the local chromatin topology is a new layer of the regulation of gene expression [114,116].

## Databases of plant lncRNAs

Mammalian lncRNAs, especially human and mouse lncRNAs, were recorded elaborately in public databases [117,118]. In addition to basic annotation information, the expression level and imprinting information of mammalian lncRNAs were also deposited in specific databases [17,119,120]. Unlike mammalian lncRNAs, lncRNAs identified in plants were not comprehensively and timely recorded in public databases. Currently, only six databases are available for depositing plant lncRNAs. These include TAIR—*Arabidopsis* gene structure and function annotation [121], PlantNATsDB—a comprehensive database of plant NATs [122], lncRNAdb—a reference database for lncRNAs [123,124], NONCODE—integrative annotation of lncRNAs [125,126], PLncDB—plant lncRNA database [8], and PNRD—a plant ncRNA database [127]. The functions, features, and links to these databases are listed in Table 2. Among these databases, NONCODE and lncRNAdb are comprehensive databases but not specifically designed for recording plant lncRNAs. PlantNATsDB deposits about 2 million NAT pairs from 70 plant species, but providing no genomic view. Although initially designed for plants, PLncDB currently deposits lncRNA information only of *Arabidopsis*, and aims to contain comprehensive information including genomic, transcriptomic, and epigenomic information related to plant lncRNAs. PNRD aims to provide lncRNAs of 150 plant species and now contains 5571 lncRNAs of *A. thaliana*, *Oryza sativa*, *Zea mays*, and *Populus trichocarpa* only.

## Concluding remarks and future perspectives

The rapid development of high-throughput RNA-seq and related bioinformatics methods provides revolutionary ways for discovering novel lncRNAs [39,128]. In recent years, many more lncRNA transcripts have been identified. Their number and types are far beyond previous expectations. lncRNA studies have become one of the new hotspots in current molecular biology. However, compared to the studies on humans and animals, the research in plants is still premature [25,129].

lncRNAs reported in plant species are limited to only a few model angiosperm plants such as *Arabidopsis*, rice, maize, wheat, foxtail millet, and soybean [7,13,27,28,40,42–44,46,130]. The task of discovering new plant lncRNAs is still very arduous. In recent years, DNA sequencing of the plant genome has developed rapidly, and genome sequencing data of dozens of plant species have been reported [131]. However, the annotations of most of the plant species lack information of lncRNAs. With the improved quality of plant genomic sequences, discovery of new lncRNAs will be more thorough and convenient. Many novel lncRNAs will be identified in plants with the increasingly-sophisticated high-throughput sequencing technology, especially strand-specific RNA-seq technology [132].

Functional studies on plant lncRNAs are a challenge. As described in this review, the current functional studies in plants are confined to a few cases [22,86,89]. Compared to protein-coding genes, mutants corresponding to lncRNAs are rare and not easy to be identified, which poses difficulties for functional studies. Systematic discovery and identification of mutant plants will help resolve the biological functions of lncRNAs. A recent endeavor in functional identification and prediction of novel lncRNAs in rice on a large scale by screening of a mutant library has been a promising example [44]. Besides, the traditional reverse genetics, such as over-expression and RNAi, as well as the lately popular CRISPR/cas9 genome editing technology [133], may also play their roles in promoting functional analysis.

## Competing interests

The authors have declared that no competing interests exist.

## Figures and Tables

**Table 1:** Summary of the lncRNAs reported in plants

**Name**	**Species**	**Biological function**	**Regulation mechanism**	**Refs.**
*COLDAIR*	Arabidopsis (*Arabidopsis thaliana*)	Flowering time	Histone modification	[22]
*COOLAIR*	Arabidopsis (*Arabidopsis thaliana*)	Flowering time	Promoter interference	[23,77,78]
*LDMAR* (*P/TMS12-1*)	Rice (*Oryza sativa*)	Fertility	Promoter methylation	[82–84]
*HID1*	Arabidopsis (*Arabidopsis thaliana*)	Photomorphogenesis	Chromatin association	[86]
*IPS1*	Arabidopsis (*Arabidopsis thaliana*)	Phosphate homeostasis	Target mimicry	[61]
*Cis-NAT_PHO1;2_*	Rice (*Oryza sativa*)	Phosphate homeostasis	Translational enhancer	[89]
*OsPI1*	Rice (*Oryza sativa*)	Phosphate homeostasis	Unknown	[95]
*TPS11*	Tomato (*Solanum lycopersicum*)	Phosphate homeostasis	Unknown	[96]
*asHSFB2a*	Arabidopsis (*Arabidopsis thaliana*)	Vegetative and gametophytic development	Antisense transcription	[97]
HvCesA6 lnc-*NAT*	Barley (*Hordeum vulgare*)	Cell-wall synthesis	siRNA precursor	[98]
SHO lnc-*NAT*	Petunia (*Petunia hybrida*)	Local cytokinin synthesis	dsRNA degradation	[99]
*GmENOD40*	Soybean (*Glycine max*)	Nodule formation	Protein re-localization	[102]
*OsENOD40*	Rice (*Oryza sativa*)	Nodule formation	Unknown	[104]
*MtENOD40*	Barrel medic (*Medicago truncatula*)	Nodule formation	Protein re-localization	[107]
*ASCO-lncRNA*	Arabidopsis (*Arabidopsis thaliana*)	Lateral root development	Alternative splicing regulators	[111]
*APOLO*	Arabidopsis (*Arabidopsis thaliana*)	Auxin-controlled development	Chromatin loop dynamics	[114]

*Note:* COLDAIR, cold assisted intronic noncoding RNA; COOLAIR, cold induced long antisense intragenic RNAs; LDMAR, long day-specific male-fertility-associated RNA; HID1, hidden treasure 1; IPS1, induced by phosphate starvation 1; PHO1;2, PHOSPHATE1;2; PI1, phosphate-limitation inducible gene 1; OsPI1, *Oryza sativa* phosphate-limitation inducible gene 1; TPS11, tomato phosphate starvation-induced gene; asHSFB2a, natural long non-coding antisense RNA of heat stress transcription factor B; CesA6 lncNAT, natural antisense of CesA6 cellulose synthase gene; SHO, an enzyme responsible for the synthesis of plant cytokinins; ENOD40, early nodulin 40; ASCO, alternative splicing competitor; APOLO, auxin-regulated promoter loop.

**Table 2:** Summary of databases depositing plant lncRNAs

**Name**	**Main features**	**Link**	**Refs.**
TAIR	The *Arabidopsis* Information Resource; serves as a comprehensive data repository; multiple analysis tools available	https://www.arabidopsis.org/	[121]

PlantNATsDB	Plant NATs database; contains NATs of 70 plant species; provides prediction of NATs; deposits networks formed by NATs; GO annotation and gene set analysis available	http://bis.zju.edu.cn/pnatdb/	[122]

lncRNAdb	A reference database for lncRNAs; deposits all known functional lncRNAs and manual annotation information of lncRNAs; sequence analysis tools available	http://www.lncrnadb.org/	[123,124]

NONCODE	An integrated knowledge database of ncRNAs; deposits all kinds of ncRNAs except tRNAs and rRNAs; all sequences information were confirmed manually; provides expression profile of lncRNA genes by graphs; provides an ID conversion tool from RefSeq or Ensembl ID to NONCODE ID and a service of lncRNA identification	http://www.noncode.org/	[125,126]

PLncDB	A plant lncRNA database; currently just contains *Arabidopsis* lncRNAs; provides genome browser of lncRNAs	http://chualab.rockefeller.edu/gbrowse2/homepage.html	[8]

PNRD	A plant ncRNA database; aims to provide information of both sRNAs and lncRNAs for 150 species; multiple analysis tools available	http://structuralbiology.cau.edu.cn/PNRD/	[127]

*Note:* TAIR, The *Arabidopsis* Information Resource; PlantNATsDB, Plant Natural Antisense Transcripts DataBase; lncRNAdb, A reference database for lncRNAs; NONCODE, An integrated knowledge database of ncRNAs; PLncDB, A plant lncRNA database; PNRD, A plant ncRNA database.
